# Transverse V-Y advancement composite tissue flap for repairing defects after longitudinal melanonychia excision—a retrospective cohort study

**DOI:** 10.3389/fsurg.2025.1575700

**Published:** 2025-04-23

**Authors:** Jianhua Zhang, Zhenjun Xie, Wei Su

**Affiliations:** ^1^ Department of Orthopaedics Trauma and Hand Surgery, The First Affiliated Hospital of Guangxi Medical University, Nanning, Guangxi, China; ^2^ Department of Hand and Foot Microsurgery, Henan Provincial People's Hospital, Zhengzhou University, Zhengzhou, Henan, China

**Keywords:** longitudinal melanonychia, nail surgery, V-Y flap, repairing defects, retrospective cohort study

## Abstract

Longitudinal melanonychia (LM) is a common nail disorder that sometimes requires surgical excision to rule out malignancy. However, longitudinal complete removal of LM, as one type of method for some special patient, can leave a significant defect in the nail bed. We introduced and assessed the application of transverse V-Y advancement composite tissue flap, a novel surgical procedure designed to address these defects. From September 2017 to January 2024, a total of 17 patients with LM underwent complete excision of the affected nail bed. The tissue defects ranged from 3 to 8 mm in width. The wounds were repaired using the transverse V-Y advancement composite tissue flap, which included the nail, nail fold, and adjacent finger pulp skin. All patients were followed up to assess flap survival, nail regrowth, and functional outcomes, and the mean follow-up time was 14 months, with a range of 6–23 months. All 17 flaps survived without complications. Nail regrowth was satisfactory in 15 cases, with only 2 cases showing a slight longitudinal ridge. Sensory recovery was well in all patients. Recurrence of LM was observed in 1 case (5.9%) during the follow-up period. The transverse V-Y advancement composite tissue flap is a reliable and effective alternative for repairing defects after LM complete excision identified as the indicating lesion, particularly for defects ranging from 3 to 8 mm in width. This technique significantly improves both functional and cosmetic outcomes, with high patient satisfaction.

## Introduction

Longitudinal melanonychia (LM) is a common lesion in hand and foot or dermatologic surgery clinic. Although most of them are benign lesions, the existence of a monodactylic longitudinal pigmented streak always evokes the possibility of a nail melanoma (1). Several clinical criteria are known to be highly suggestive of melanoma (2, 3). Dermatoscopic criteria have been added (4), but histology remains the gold standard for diagnosis, and surgical excision remains the most effective approach for managing this condition (1, 2, 5, 6).

**Figure 1:**
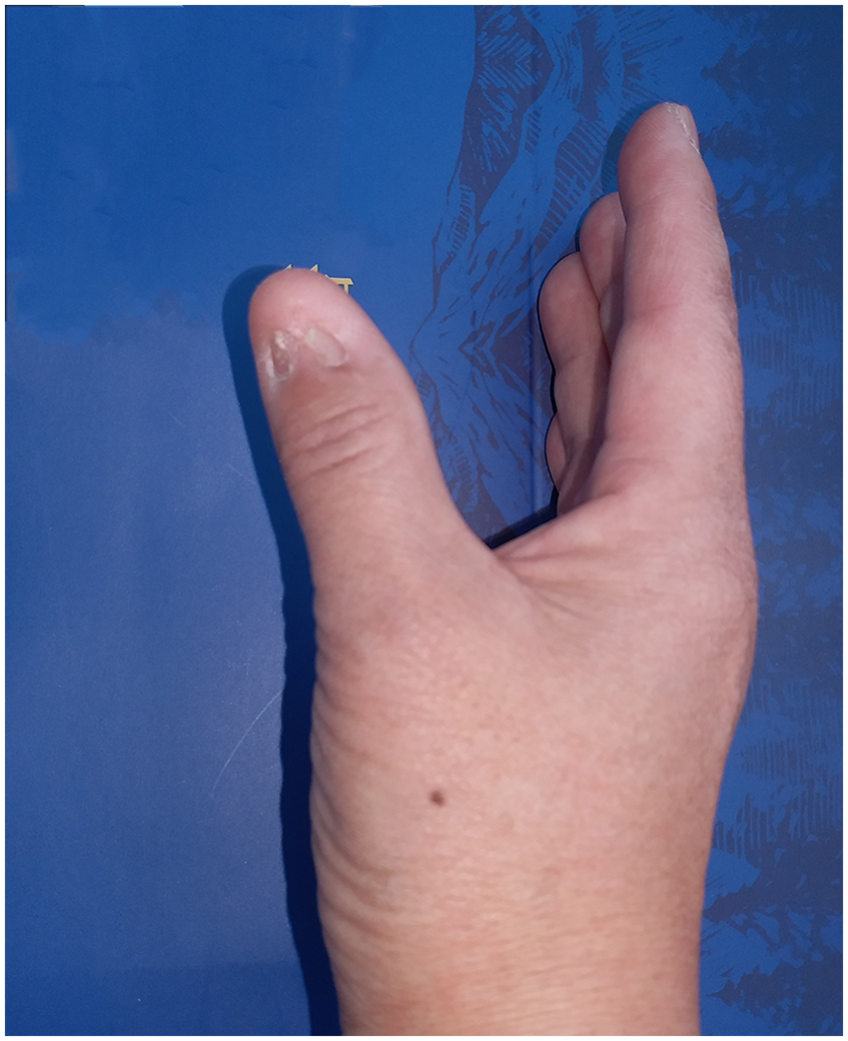
Appearance after skin graft operation.

Several surgical techniques are available for the treatment of LM, including the double punch technique, transverse matrix technique, tangential matrix excision and longitudinal complete excision. Each techniques suits different LM types, and longitudinal complete excision is one of the essential methods (2, 7). This technique offers several advantages: it allows for the collection of a complete specimen, enhancing the accuracy of pathological diagnostic, and facilitates thorough removal of the lesion, reducing the risk of pigmentation recurrence. Indications for this technique primarily encompass periungual pigmentation or pigmentation of the nailfold, lesion greater than 3 mm located at proximal nail matrix, Hutchinson's sign, etc. However, wound repair after *en bloc* excision is challenging when the defect exceeds 3 mm in width. In such cases, repair typically requires either skin graft, or U- flap technique (2, 8). However, the aesthetic outcome with skin grafts is often unsatisfactory, as the nail may be divided into two parts (Figure 1). Meanwhile, although the U-flap technique is effective, it can leave a secondary wound, leading to increased pain and prolonged healing times for patients.

To improve the result of LM that require longitudinal complete excision, we propose a novel approach to manage the defect after LM excision by using transverse V-Y advancement composite tissue flap and present the results of its application in series cases.

## Patients and methods

From September 2017 to January 2024, a total of 17 patients (7 males and 10 females) with LM were admitted to our department. The patients' ages ranged from 3 to 57 years, with a mean age of 41.4 years. Among all the patients, 12 had LM on their fingers (4 on thumbs and 8 on other fingers), while 5 had LM on their toes (3 on the big toes and 2 on other toes), the width of tissue defect after LM excision varied from 3 to 8 mm. Our inclusion criteria for this study included: (1) LM with presence of periungual pigmentation or pigmentation of the nailfold; (2) lesion greater than 3 mm located at proximal nail matrix; (3) hutchinson's sign; and (4) patients with a strong desire for surgical treatment. Our exclusion criteria included: (1) lesion less than 3 mm located at distal nail matrix; (2) lesion greater than 8 mm exceeding the advancement capability of the V-Y flap; (3) LM with malignant potential requiring amputation.

## Surgical technique

After surgical disinfection, a transthecal anesthesia was performed for complete anesthesia of the nail apparatus. Expanded excision was performed by 1 mm on both sides of the LM. And the affected nail, nail bed, nail matrix and proximal nail fold were removed, until the bone periosteum was exposed.

**Figure 2:**
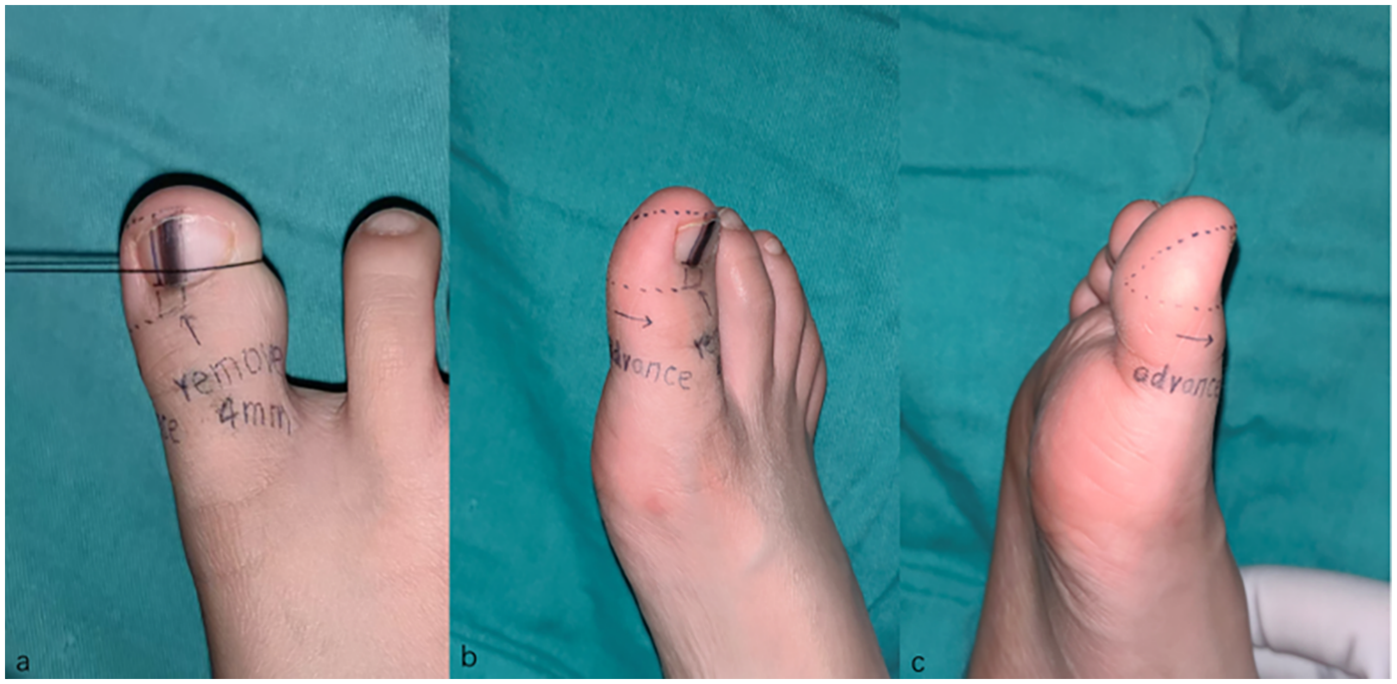
Design of the transverse V-Y advancement composite tissue flap.

**Figure 3:**
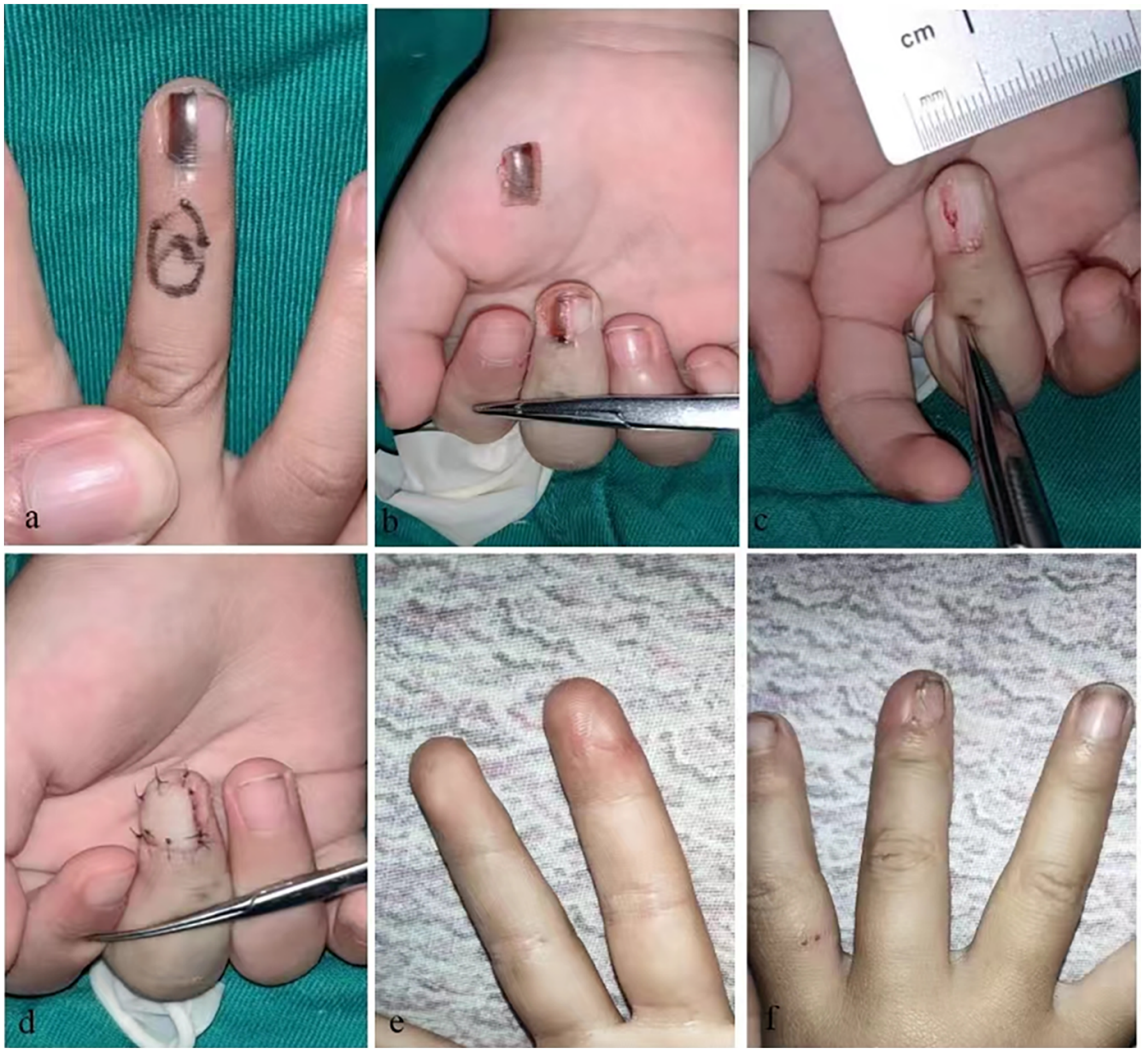
**(a)** Longitudinal melanonychia on middle finger. **(b)** LM involve pigmentation of the nail bed, nail matrix, and periungual epithelium. **(c)** LM was completely removed. **(d)** A advancement triangle-shaped composite tissue flap was harvested to cover the defect. **(e,f)** Follow-up image showing the appearance of the finger.

A right triangle-shaped composited tissue flap was outlined on the side where smaller area of intact nail apparatus remained. After incision along the marked line, the lateral cutaneous ligament was cut off and the continuity of the vessels and nerves on the palm side was well preserved. Then the advancement flap was advanced towards the dorsal midline to cover the defect. However, when the defect is larger than the coverage range of the traditional V-Y flap, the underneath of the advancement flap can be undermined and only the vascular nerve bundle of the palm side is retained. Nail bed, nail matrix and nail fold were then accurately aligned and sutured with 6–0 absorbable suture. The secondary defect in the ventral side was sutured directly (Figures 2, 3).

At the final follow-up after surgery, the appearance of each patient's fingers and nails, as well as their sensation, were assessed, and patients were asked about their satisfaction with their fingers. We used a semi-quantitative method, dividing satisfaction into four levels: very satisfied, satisfied, neutral, and dissatisfied, and recorded each patient's response. For younger patients, we assessed treatment satisfaction primarily by interviewing their parents or guardians.

## Results

In the 17 patients who underwent transverse V-Y advancement composite tissue flap coverage, all grafted flaps survived well. Pathological diagnosis was successfully obtained in every case, revealing melanocytic activation in six cases, lentigo in five cases, and nevus in six cases (five junctional and one compound). The nail plate grew well in all patients, with slight longitudinal ridging observed in two cases. Sensory function was well restored in the advancement flaps. The regenerated nails showed no signs of dystrophy. All cases were followed up, and the mean follow-up time was 14 months, with a range of 6–23 months, during which recurrence was observed in one case at the same site. The recurrent case underwent another surgery with an expanded resection of the lesion. The wound was closed with a small skin graft and healed, but the nail appearance was less than optimal. In the 17 patients, most patients (15) expressed satisfaction (9) or very satisfaction (6) with the post-treatment appearance and functional recovery, while a few (2) were neutral (1) or dissatisfied (1).

## Typical case

**Figure 4:**
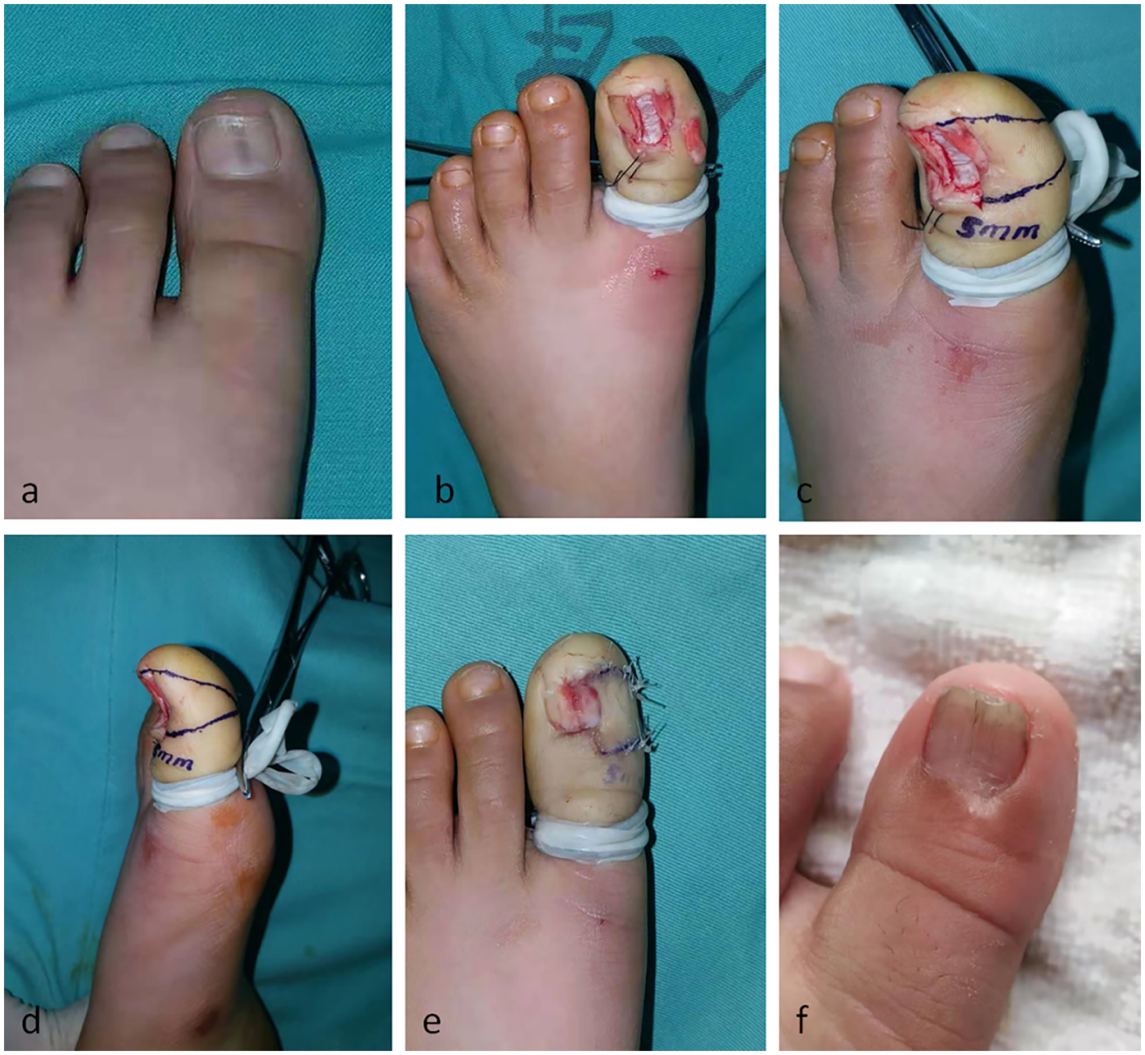
**(a)** Longitudinal melanonychia on hallux. **(b)** LM was completely removed. **(c)** A right triangle-shaped skin flap was outlined on the right side of the defect. **(d)** The lateral view of flap design. **(e)** the flap was advanced to cover the defect. **(f)** 1-year follow-up image shows a good appearance of nail plate, being smooth and flat, with no longitudinal ridges.

An 18-year-old male patient presented with a band of longitudinal melanonychia affecting his left great toe. Physical examination revealed a 4-mm band of longitudinal melanonychia on the left great toe, which had gradually progressed over the past month (Figure 4).

After surgical disinfection, transthecal anesthesia was performed to achieve complete anesthesia of the nail apparatus. An expanded excision was carried out, extending 1 mm on both sides of the longitudinal melanonychia (LM). The affected nail, nail bed, nail matrix, and proximal nail fold were removed *en bloc*. A right triangle-shaped skin flap was outlined on the right side. After incision along the marked line, the lateral cutaneous ligament was severed. The advancement flap was then pushed towards the dorsal midline to cover the defect area. The nail bed, nail matrix, and nail fold were accurately sutured with 6–0 absorbable sutures. The secondary defect on the ventral side was directly sutured. The postoperative pathological results showed a junctional nevus.

After a 1-year follow-up, the nail of the left great toe had a good appearance, being smooth and flat, with no longitudinal ridges on the nail plate. The patient was satisfied with the postoperative appearance of the toe, and the sensory function in the advancement flap had recovered well. The regenerated nail showed no signs of dystrophy, and no recurrence was observed.

## Discussion

Longitudinal melanonychia (LM) is a common condition in dermatology and hand and foot clinics, characterized by a longitudinal pigmented band extending from the nail matrix to the distal part of the nail plate due to melanin deposition. Melanin is produced by melanocytes in the matrix and incorporated in the nail plate (5, 8, 9). Despite the many years of development in its diagnosis and treatment (2, 4), histology remains the gold standard for accurate diagnosis, and surgery excision is the primary treatment modality (1, 2, 5, 6). Surgery techniques (4, 6) for LM include double punch biopsy (recommended for pigmented bands <3 mm originating from the distal matrix), transverse matrix biopsy (suitable for bands >3 mm from the distal matrix), and tangential matrix excision (indicated for low-suspicion lesions >3 mm in the middle of the nail or originating from the proximal matrix). However, tangential matrix excision is associated with a high recurrence rate of pigmentation (70%) (9–11). Therefore, longitudinal complete excision, which involves the removal of the entire nail apparatus (including the nail plate, nail bed, nail matrix, and proximal nail fold), is sometimes necessary, especially in cases with periungual pigmentation, lesions >3 mm in the proximal nail matrix, or Hutchinson's sign. This technique improves diagnostic accuracy and reduces the recurrence of pigmentation (1–3, 8, 10, 11).

However, the repair of the wound after *en bloc* excision remains a challenge, regardless of whether your surgical procedure is intended for pathological examination of the lesion or for complete excision. When the width of the resulting defect is less 3 mm, direct suturing is feasible. In contrast, when the defect width exceeds 3 mm, more complex techniques such as skin graft, or the U- flap are required. However, the former technique may result in less satisfactory cosmetic outcomes, particularly when the defect is located in the mid-portion of the nail, as it may leave the nail divided into two parts (as shown in Figure 1). On the other hand, U-flap reconstruction can provide better aesthetic results but come with the drawback of leaving a secondary wound, which can cause more pain for the patient and require a longer healing time.

V-Y flaps, often hailed as “local flaps that never disappoint”, are renowned for their numerous advantages and ease of operation. Traditionally, V-Y flaps on the palm or the palmar surface of the finger are primarily used to repair skin and soft tissue defects on the volar aspect of the finger or palm, with limited application for defects on the dorsal finger or nail bed (12, 13). To address the challenges of repairing nail bed defects described earlier, we have introduced a flexible application of the V-Y flap. Compared to conventional methods, this approach represents a viable alternative. It effectively resolves the issue of repairing midline nail bed defects and largely avoids the deformity of nail division. The outcomes in most cases have been highly satisfactory for both patients and surgeons. The surgical procedure is relatively straightforward, eliminating the need for skin grafts from distant donor sites. Moreover, the postoperative appearance is significantly enhanced. Patients who undergo this surgery retain a complete nail, albeit slightly smaller, which is far superior to a divided, two-piece nail.

Width of nails varies among individuals and on different fingers and toes. For instance, the nail width differs between adults and children, between the thumb and the little finger, and between fingers and toes. Given this variability, it is practical to describe the width of nail defects using ratios rather than absolute measurements. For patients with a longitudinal melanonychia (LM) excision defect that is smaller than one-third of the nail width, the transverse V-Y advancement composite tissue flap repair can achieve satisfactory outcomes in both appearance and function. For those with a defect that is larger than one-third but smaller than two-thirds of the nail width, this method can still result in significant aesthetic improvement, even though a smaller portion of the nail is retained. Importantly, this approach eliminates the need for a skin graft in the middle of the nail, which is often a source of discomfort for patients. However, for patients with an LM excision defect that exceeds two-thirds of the nail width, alternative methods such as skin graft repair or other types of flaps should be considered.

Several tips should be closely followed during the surgery procedure: (1) The nail bed is tightly combined with the distal phalanx and lateral skin. To preserve the integrity of the nail bed, nail matrix, and nail fold, and to maintain the blood circulation system, it is recommended to perform subperiosteal undermining of the nail. This approach ensures that the regenerated nail has a better appearance. (2) The advancement flap, which includes the nail bed, matrix, and nail fold, should be precisely integrated with the remaining tissue. Therefore, it is highly recommended to perform the entire suturing process of the nail bed and nail matrix under a microscope to achieve optimal precision and outcomes. Our technique is suitable for most cases, as the width of the lesion in most patients ranges from 3 to 8 mm.

In clinical practice, decisions regarding whether to operate and which surgical approach to use for managing LM remain challenging for both patients and surgeons. While some of the cases presented in this paper may be considered controversial from a dermatologist's perspective, the primary focus of this study is to introduce an alternative surgical method for repairing defects following LM excision. This method is particularly beneficial for cases that are deemed suitable for longitudinal excision of the lesion.

## Conclusion

The transverse V-Y advancement composite tissue flap to repair wounds after the longitudinal complete excision of LM identified as the indicating lesion is a satisfactory surgical technique for function and appearance recovery, providing another alternative for surgeons.

## Data Availability

The raw data supporting the conclusions of this article will be made available by the authors, without undue reservation.
